# Impact of anterior commissure involvement on recurrence in early-stage vocal cord tumors: a propensity score analysis

**DOI:** 10.1007/s00405-025-09357-1

**Published:** 2025-04-07

**Authors:** Carlos Galán García-Hortelano, Javier Gavilanes Plasencia, Alfred García Fernandez

**Affiliations:** 1 Lister Hospital., East and North Hertfordshire NHS Trust, Stevenage, United Kingdom; 2 Hospital U. 12 de Octubre, Madrid, Spain

**Keywords:** Propensity score, Anterior commissure, TOLMS, Recurrence, TNM, Observational study, Confounding variables

## Abstract

**Background:**

Laryngeal cancer is one of the most common head and neck tumors, with 75% affecting the vocal cords. The 8th edition of the TNM staging system defines T1 glottic tumors as those limited to the vocal cords with preserved mobility. Since the publication of the third edition in 1998, this category has been divided into T1a (tumor limited to one vocal cord) and T1b (both vocal cords involved). However, these tumors can also involve the anterior commissure. The anterior commissure is considered a sublocation by the American Joint Committee on Cancer (AJCC) and the Union for International Cancer Control, but it is not accounted for in the current T staging system. Although the anterior commissure is rarely the primary site of glottic tumors (1%), 20% of glottic tumors show involvement of the anterior commissure, with its impact on prognosis still controversial.

**Methods:**

A global and specific survival analysis was performed using the Kaplan-Meier method, comparing survival curves with the Log Rank test. A Cox regression model was constructed, including confounding variables and examining possible interaction terms, evaluating the proportionality assumption through graphical methods. Confounding variables were controlled using the Propensity Score (PS), estimating the effect with different PS methods.

**Results:**

The variable “Anterior Commissure” showed a significant effect on the recurrence of glottic cancer, consistent across the different propensity score adjustment methods. The Inverse Probability of Treatment Weighting (IPTW) method was particularly effective in adjusting for covariate differences between groups, maintaining the full sample size, and providing a robust and clinically relevant analysis.

**Conclusions:**

The anterior commissure is a significant risk factor for the recurrence of glottic cancer. Integrating propensity score methods enhances the precision and validity of survival studies. It is recommended to continue exploring these methods in larger and more diverse cohorts.

## Introduction

Laryngeal carcinoma is one of the most prevalent malignancies in the head and neck region, with approximately 157,000 new cases diagnosed annually, accounting for about 1.1% of all tumors worldwide [1]. Glottic tumors, specifically those affecting the vocal cords, constitute roughly 75% of all laryngeal carcinomas [2–5]. The American Joint Committee on Cancer (AJCC) and the Union for International Cancer Control classify the glottis into three sublocations: vocal cords, anterior commissure, and posterior commissure [6]. However, the eighth edition of the TNM staging system defines T1 glottic tumors as those confined to the vocal cords with preserved mobility [7]. Since the third edition of the TNM system in 1988 [8], the T1 category has been further subdivided into T1a (tumors limited to one vocal cord) and T1b (tumors involving both vocal cords).

Primary tumors arising from the anterior commissure are rare, comprising only 1% of laryngeal cancers. Nevertheless, approximately 20% of glottic tumors exhibit anterior commissure involvement, and its prognostic significance remains a subject of debate [9–11]. Treatment modalities for early-stage glottic cancer include transoral laser microsurgery (TOLMS), radiotherapy (RT), and, in selected cases, open surgery. Due to its invasive nature and higher morbidity, open surgery is generally not recommended as the first-line management for T1 glottic tumors6 [10, 12–16], it is considered only when less invasive options are unsuitable. Consequently, the role of open surgery in early-stage glottic carcinomas has markedly diminished over the past decade [2, 13, 17].

The prognostic impact of anterior commissure involvement in early-stage glottic tumors remains unclear. Therefore, this study aimed to evaluate the overall and disease-specific survival of T1N0 vocal cord tumors treated at a tertiary healthcare institution in the Community of Madrid using propensity score analysis, with a focus on determining the impact of anterior commissure involvement on 5-year and 10-year recurrence outcomes.

## Methods

### Study design

A retrospective cohort study was conducted involving patients treated between January 1, 2010, and January 1, 2024. Data were obtained from the Otolaryngology department at Hospital Universitario 12 de Octubre.

### Inclusion and exclusion criteria

Eligible patients included adults with histopathologically confirmed T1N0 glottic carcinoma classified according to the TNM classification system. Patients with incomplete information regarding tumor sublocation within the vocal cords or a history of previous laryngeal surgeries or radiotherapy were excluded from the study.

### Variables

The primary exposure variable was tumor sublocation, specifically anterior commissure involvement. Additional demographic data, such as age and sex were recorded, along with established prognostic factors, including TNM staging, treatment modality (19 patients were treated with radiotherapy and 49 with transoral laser microsurgery), exposure to tobacco and alcohol, and histological differentiation. The primary outcome was the recurrence of glottic carcinoma.

### Preoperative assessment an follow-up

At our center, the routine preoperative examination included magnifying laryngoscopy and phoniatric evaluation, with no differences between patients with anterior commissure involvement and those without, given the early stage of the tumors. Patients were seen 10 days after surgery to discuss the histopathology results and the treatment plan established in the multidisciplinary team (MDT) meeting. Follow-up visits were scheduled every 3 months during the first year, every 6 months from the first to the third year, and annually from the third to the fifth year, after which patients were discharged.

### Statistical methods

All statistical analyses were conducted using R, version 4.3.3 (R Core Team, 2024), with a 5% significance level (alpha) and a power of 80% (beta). Quantitative variables were summarized using mean and standard deviation or median and interquartile range, as appropriate. Categorical variables were summarized using proportions and 95% confidence intervals (CI).

Initially, univariate analyses were performed to assess the associations between potential prognostic factors and the outcome of interest. For continuous variables, Student’s t-test was used, while Chi-square or Fisher’s exact tests were applied to categorical variables. Subsequently, a multivariate Cox proportional hazards regression model was developed to adjust for potential confounders and evaluate the independent impact of anterior commissure involvement on recurrence.

Following the multivariate modeling, propensity score (PS) analysis was performed to further control for confounding by estimating the probability of anterior commissure involvement based on baseline characteristics. Different PS-based methodologies were applied, including matching, stratification, and inverse probability weighting (IPW). This allowed for additional adjustment of confounders and assessment of the robustness of the observed associations.

## Results

A descriptive analysis of the dataset included 68 patients (61 males, 7 females) who met the inclusion criteria, with a mean age of 70 years (SD: 11.94 years). To summarize the patient characteristics (see Table 1 for detailed distribution by stage, recurrence and anterior commissure involvement):

**Table 1:** Patient distribution by tumor stage, recurrence and anterior commissure involvement

Category	With Anterior Commissure	Without Anterior Commissure	Total
In situ stage	-	7	7
Stage T1a	10	35	45
Stage T1b	12	4	16
Recurrence	12	7	19
No Recurrence	12	37	49

Recurrence was observed in 19 patients, with anterior commissure involvement present in 12 of these cases. Among the 49 patients without recurrence, 12 had anterior commissure involvement, while 37 did not.

Specifically, recurrence occurred in 13 patients with T1a tumors, including in situ, and 6 patients with T1b tumors. Among the 49 patients without recurrence, 39 had T1a tumors, also including in situ, and 10 had T1b tumors (see Table 2 for recurrence distribution by tumor stage).

**Table 2:** Distribution of patients by tumor stage and recurrence status

Stage	Recurrence	No Recurrence
T1a (including in situ)	13	39
T1b	6	10

In most cases, patients with recurrences were treated with repeat TOLMS when feasible (12 patients). For more advanced recurrences or cases where TOLMS was not viable, radiotherapy (5 patients) or radical surgery (2 patients) were considered.

No major postoperative complications were reported. No tracheotomy or feeding tube was required, and no late complications occurred.

No significant differences were observed between patients with and without anterior commissure involvement regarding baseline characteristics such as age, sex, tobacco exposure, or alcohol consumption. Additionally, 19 patients were treated with radiotherapy and 49 with transoral laser microsurgery, with no statistically significant differences observed in the univariate analysis.

**Fig. 1:**
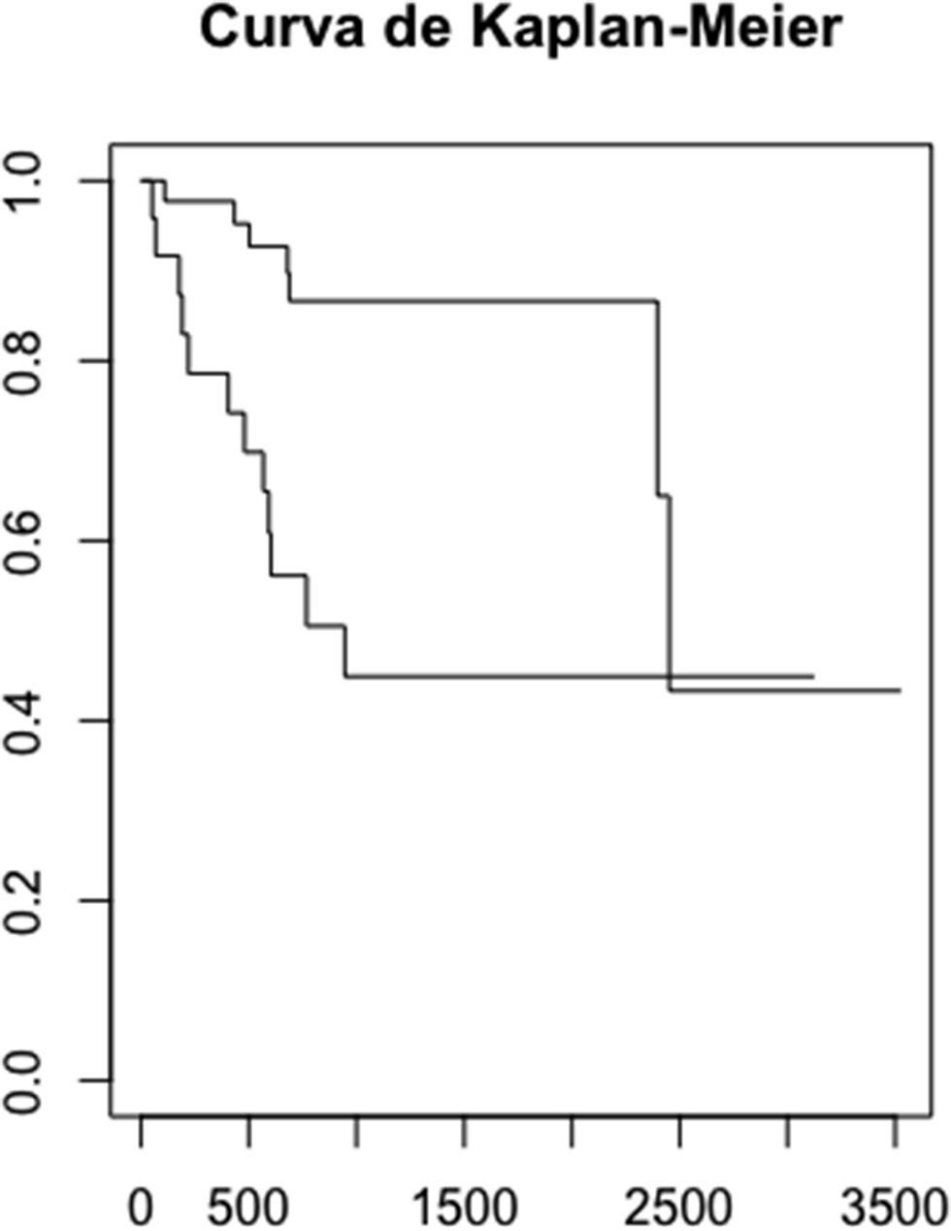
Kaplan-Meier curve for the anterior commissure involvement

Kaplan-Meier survival analysis demonstrated a significant difference in disease-free survival between the two groups (log-rank *p* = 0.03). Specifically, the median disease-free survival for patients without anterior commissure involvement was longer compared to those with involvement. The mean disease-free survival time for patients with anterior commissure involvement was approximately 20 months, whereas for patients without involvement, it was approximately 34 months (Fig. 1).

In contrast, no significant difference in disease-free survival was found when comparing Radiotherapy and TOLMS (log-rank *p* = 0.2).The Kaplan–Meier analysis indicated similar survival outcomes for both treatment groups in this cohort.

In the multivariate Cox proportional hazards model, anterior commissure involvement was independently associated with an increased risk of recurrence (HR: 4.09, *p* = 0.003), after adjusting for potential confounders.

Different methods of propensity score analysis were used to further validate these findings, including matching, stratification, inverse probability weighting (IPW), and covariate adjustment. The hazard ratios (HR) for anterior commissure involvement were consistently high across these methods: 3.629 for propensity score matching (95% CI: 1.16–11.36), 3.226 for stratification (95% CI: 1.175–8.858), 3.531 for IPW (95% CI: 1.342–9.294), and 4.094 for covariate adjustment (95% CI: 1.569–10.68). Each of these methods showed a significant increase in the risk of recurrence (*p* < 0.05), reinforcing the robustness of the findings (Table 3).

**Table 3:** Comparison of hazard ratios by propensity score method

PS Method	Hazard Ratio (HR)	95% CI
Matching	3.629	(1.16, 11.36)
Stratification	3.226	(1.175, 8.858)
IPTW Weighting	3.531	(1.342, 9.294)
Covariate Adjustment	4.094	(1.569, 10.68)

## Discussion

The results of this study indicate that anterior commissure involvement is a significant predictor of recurrence in early-stage glottic carcinoma [18–20]. The unique anatomical characteristics of the anterior commissure may contribute to this increased risk, as they can facilitate tumor extension and complicate complete resection [2, 4, 18–21]. This aligns with previous research linking anterior commissure tumors to worse local control due to complex anatomy and limited accessibility [2–4, 6, 18–21].

Higher recurrence rates in patients with anterior commissure involvement call for careful treatment planning and more rigorous monitoring. Moreover, the mean disease-free survival time was significantly shorter: 20 months for those with involvement compared to 34 months for those without. These findings highlight the importance of tailored follow-up strategies to ensure early detection and timely treatment of recurrences. At our center, local recurrences were managed based on the clinical features and extent of the recurrence.

From all PS methods, the inverse probability of treatment weighting (IPTW) was chosen for our survival analysis due to its robustness, ability to utilize the full sample, and simplicity. IPTW uses all data points, maximizing statistical power and ensuring representativeness. By weighting observations inversely to their treatment probability, IPTW effectively balances covariates, leading to a more accurate estimation of the impact of anterior commissure involvement. Additionally, IPTW produced a significant hazard ratio consistent with other propensity score methods, reinforcing its reliability while maintaining transparency and ease of interpretation.

The hazard ratio of 3.5 from IPTW propensity score analysis means that patients with anterior commissure involvement have a 3.5 times higher risk of recurrence compared to those without. Despite the wide confidence interval (95% CI: 1.6–10.7), which suggests some uncertainty due to sample size limitations or group imbalances, the consistent hazard ratio across different methods still points to a robust association between anterior commissure involvement and increased recurrence risk [21–26].

The consistency across various propensity score methodologies—including matching, stratification, IPTW, and covariate adjustment—reinforces the reliability of the findings. The hazard ratio for anterior commissure involvement remained stable across all models (HR: 3.2–4.1), highlighting its role as a significant prognostic factor for recurrence. The multivariate Cox model demonstrated an elevated risk (HR: 4.09, *p* = 0.003). This aligns with the study by Piazza in March 2018, which analyzed 410 patients (284 with T1a and T1b tumors) and reported a similar outcome in early-stage tumors with anterior commissure involvement (HR: 4.0, *p* = 0.001), emphasizing that vertical extension of the anterior commissure should be considered a major weakness for TOLMS [26].

One limitation of this study is the potential overestimation of the recurrence rate due to the exclusion of patients without specific information on tumor sublocation. It is likely that when the tumor location was not detailed, it did not involve critical structures such as the anterior commissure. As a result, these patients were excluded, potentially leading to an overrepresentation of cases with anterior commissure involvement and, therefore, a higher recurrence rate.

Further studies are required to confirm the impact of anterior commissure involvement on prognosis, refine treatment protocols, and develop follow-up strategies for this subgroup of patients. Findings consistently demonstrated that anterior commissure involvement is associated with a significantly increased risk of recurrence, regardless of the methodology used for controlling confounding variables, emphasizing its prognostic relevance. Efforts must be made to reduce recurrence rates and improve patient outcomes.

## Conclusion

Involvement of the anterior commissure in early-stage glottic cancer is linked to a higher risk of recurrence, making it an important factor in predicting patient outcomes. This means that more personalized treatment and closer monitoring might be needed for these patients. More research is needed to find the best treatment approaches for those with anterior commissure involvement.
