# Correction: Impact of anterior commissure involvement on recurrence in earlystage vocal cord tumors: a propensity score analysis

**DOI:** 10.1007/s00405-025-09720-2

**Published:** 2025-10-11

**Authors:** Carlos Galán García-Hortelano, Javier Gavilanes Plasencia, Alfred García Fernandez

**Affiliations:** 1 Lister Hospital., East and North Hertfordshire NHS Trust, Stevenage, United Kingdom; 2 Hospital U. 12 de Octubre, Madrid, Spain

**Correction: European Archives of Oto-Rhino-Laryngology (2025) 282:4237–4241**

10.1007/s00405-025-09357-1

The article “Impact of anterior commissure involvement on recurrence in earlystage vocal cord tumors: a propensity score analysis”, written by Carlos Galán García-Hortelano, Javier Gavilanes Plasencia and Alfred García Fernandez was originally published under exclusive license to The Author(s), under exclusive licence to Springer-Verlag GmbH Germany, part of Springer Nature. As a result of the subsequent decision to publish the article under the open access model, the article’s copyright notice was changed on 31 August 2025 to © The Author(s) 2025 and the article is now distributed under a Creative Commons Attribution 4.0 International License, which permits use, sharing, adaptation, distribution and reproduction in any medium or format, as long as you give appropriate credit to the original author(s) and the source, provide a link to the Creative Commons licence, and indicate if changes were made. The images or other third party material in this article are included in the article’s Creative Commons licence, unless indicated otherwise in a credit line to the material. If material is not included in the article’s Creative Commons licence and your intended use is not permitted by statutory regulation or exceeds the permitted use, you will need to obtain permission directly from the copyright holder. To view a copy of this licence, visit http://creativecommons.org/licenses/by/4.0.

The original article has been corrected.

